# Resistance induction and nematicidal activity of certain monoterpenes against tomato root-knot caused by *Meloidogyne incognita*

**DOI:** 10.3389/fpls.2022.982414

**Published:** 2022-09-20

**Authors:** Mohsen Mohamed Elsharkawy, Abdulaziz A. Al-Askar, Said I. Behiry, Ahmed Abdelkhalek, Muhammad Hamzah Saleem, Muhammad Kamran, Aly Derbalah

**Affiliations:** ^1^Department of Agricultural Botany, Faculty of Agriculture, Kafrelsheikh University, Kafr El Sheikh, Egypt; ^2^Department of Botany and Microbiology, College of Science, King Saud University, Riyadh, Saudi Arabia; ^3^Agricultural Botany Department, Faculty of Agriculture (Saba Basha), Alexandria University, Alexandria, Egypt; ^4^Plant Protection and Biomolecular Diagnosis Department, ALCRI, City of Scientific Research and Technological Applications, Alexandria, Egypt; ^5^MOA Key Laboratory of Crop Ecophysiology and Farming System Core in the Middle Reaches of the Yangtze River, College of Plant Science and Technology, Huazhong Agricultural University, Wuhan, China; ^6^School of Agriculture, Food and Wine, The University of Adelaide, Urrbrae, SA, Australia; ^7^Pesticides Chemistry and Toxicology Department, Faculty of Agriculture, Kafrelsheikh University, Kafr El Sheikh, Egypt

**Keywords:** tomato, *Meloidogyne incognita*, resistance induction, control, monoterpenes

## Abstract

This research was performed to evaluate the potential of carvone, cuminaldehyde, cineole, and linalool for the control of root-knot of tomato. The tested control agents were evaluated for their ability to stimulate systemic resistance to *Meloidogyne incognita* in tomato by monitoring the transcription levels of defense-related genes. Moreover, the ability of the tested agents to induce nematicidal activity concerning second-stage juveniles (J2) hatching and mortality was evaluated. Furthermore, the effect of the tested agents on certain tomato growth and yield parameters was assessed. The tested monoterpenes showed high nematicidal activity against *M. incognita* concerning J2 hatching inhibition and mortality. Carvone, cuminaldhyde, linalool, and cineole had LC_50_ values of 123.5, 172.2, 354.9, 466.4, and 952.3 μg/mL, respectively. Carvone was found to be the most efficient hatching inhibitor. The tested monoterpenes showed a high potential against root-knot under greenhouse and field conditions with respect to root-galling, egg masses, and the number of J2. Carvone was the most effective treatment. The growth and yield characters of treated tomato were significantly increased in monoterpenes treatments compared to untreated control. Treated tomato plants showed expression of defense-related genes (*PR1* and *PAL*) 5-8 folds higher than the control. The results also showed that cuminaldhyde, followed by carvone, linalool, and cineole, had the greatest levels of expression in tomato plants. Taken together, the selected monoterpenes could be used as alternatives to control the root-knot of tomato.

## Introduction

Tomato (*Solanum lycopersicum* L) is an important crop worldwide. Tomato fruits are popular all over the world. Ripe tomato fruit is highly nutritious as a good source of vitamins and minerals (USDA, 2005). In addition, as a processed crop, it ranks first among vegetables (Kessel, 2003). It is consumed fresh and/or used to make pastries, mash, ketchup, and fruit drinks (Ogwulumba and Ogwulumba, 2018). Tomato is grown on various seasonal farms throughout the year in Egypt as one of the most important crops that can provide a higher income for small and large farmers than other crops (Malash et al., 2005; Abd-Elgawad, 2020).

Tomato is among the most susceptible crops to several biotic and abiotic stresses compared to other vegetables. Among the different biotic stresses, plant parasitic nematodes (PPN) are the most common and widespread pests that may cause major damage to tomato production (Abd-Elgawad, 2014, 2020; El-Shafeey et al., 2019). Plant parasitic nematodes are obligate parasites that feed on the plant. Therefore, they cause significant damage to a wide range of crops and lead to significant production losses of about $78 billion worldwide annually (Caillaud et al., 2008). The intensive use of chemical pesticides to manage PPN has resulted in environmental and health issues, as well as nematicides resistance (Molinari, 2011; Abd-Elgawad, 2020). Hence, it has become very necessary to look for alternative strategies to control PPN. Among the possible alternative strategies are the use of plant extracts, secondary plant metabolites, and plant-based essential oils (Echeverrigaray et al., 2010; Kundu et al., 2021). In nature, essential oils play an important role in the protection of plants against microorganisms, insects, and nematodes. Monoterpenoids, are the most representative molecules in more than 90% of the essential oils extracted from plants (Bakkali et al., 2008). The activity of many plant compounds and metabolites, including essential oils (mainly monoterpenes), has been reported against PPN and may provide a potential alternative to currently used nematicides (Pérez et al., 2003; Onifade et al., 2008; Echeverrigaray et al., 2010; Kundu et al., 2016; Dutta et al., 2021; Keerthiraj et al., 2021).

Resistance in crops is an important strategy for the protection from PPN (Oka et al., 2000). Plant treatments with several biotic or abiotic agents can simulate plants to resist PPN attack (Walters et al., 2005). The stimulated defense potential induced by particular environmental factors is known as induced resistance (Van Loon et al., 1998). This resistance is successful against fungi, bacteria, viruses, and PPN (Vallad and Goodman, 2004). Several low molecular weight molecules identified as phytohormones, such as salicylic acid (SA), jasmonic acid (JA), and ethylene (ET), control the immune response in plants (Vallad and Goodman, 2004). The plant’s detection of invading pathogen results in the production of signaling molecules such as phytohormones, SA, JA, and their derivatives. SA promotes resistance to biotrophic pathogens, while JA promotes resistance to necrotrophic pathogens. The induction of pathogenesis-related (PR) genes is believed to be an indicator of plant-induced defense responses against diseases attack. PR genes have been frequently used as marker genes for systemic acquired resistance in many plant species (Mitsuhara et al., 2008; Mazarei et al., 2011). In fact, only a few studies have examined the expression of the PR gene in response to root knot and cyst nematodes (Mazarei et al., 2011). Despite the fact that nematode diseases are thought to be managed, above-ground symptoms are frequently the result of below-ground infections that are difficult to detect by farmers. Nematode damage to most crops is almost always linked to the nematode’s initial numbers in the soil (Lutuf et al., 2018). Genes that give host resistance to root knot nematodes (RKN, *Meloidogyne* sp.) have been reported by Williamson and Roberts (2009) in annual and perennial crops. PR gene expression, particularly *PR-1* expression, was dramatically elevated in shoots of resistant infected plants (Vallad and Goodman, 2004). The expression of a phenylalanine ammonia-lyase (*PAL*) gene, which encodes PAL enzyme involved in the biosynthesis of the signal molecule, SA (Mauch-mani and Slusarenko, 1996). Induction of PAL activity is a reliable indicator of plant resistance expression (Mauch-mani and Slusarenko, 1996; Safaie-Farahani and Taghavi, 2017). Pathogen-infected plants have been demonstrated to produce PAL (Campos et al., 2003; Farahani et al., 2016). Plant resistance depends on PAL expression that is both more rapid and more persistent. Two non-host bacteria, *Pseudomonas syringae* pv. *phaseolicola* and *P. syringae* pv. *glycinea*, were inoculated into *Arabidopsis* plants, and an increase in PAL protein accumulation was observed (Mishina and Zeier, 2007).

The current study evaluated the *in vitro* nematicidal activity of carvone, cuminaldehyde, cineole, and linalool against *Meloidogyne incognita*, as well as their ability to control the diseases caused by the RKN in tomato plants under greenhouse and field conditions. The effect of monoterpenes on root-galling, egg masses, and the number of J2 were evaluated. Additionally, the abilities of monoterpenes to stimulate defense related-genes (*PR1* and *PAL*) expression and certain growth and yield characters of tomato were investigated.

## Materials and methods

### Chemicals

Sigma Aldrich, United States, provided the monoterpenes (carvone, cineole, cuminaldehyde, and linalool) with a purity of 99%. Oxamyl with a trade name of Vydate 310 SL produced by DuPont Company Wilmington, United States, was used as a recommended nematicide for RKN control on tomato.

### Isolation and identification of nematode

The southern root-knot nematode (*M. incognita*) was isolated from infected tomato and the adult females’ perineal patterns, as well as the morphology of second-stage juveniles (J2), were used. The isolate was reared on tomato plants Cv super strain B under greenhouse conditions (temperature at 25°C with 14 h light/10 h dark photoperiod). The roots of tomato heavily infested with pure cultures of *M. incognita* were used for the extraction of eggs, according to Hussey and Barker (1973) and Ghahremani et al. (2019). RKN egg masses were placed in sterilized distilled water (SDW) containing a sodium hypochlorite solution (0.5%) and incubated for 72 h at 25 ± 2°C for hatching. Every day, the newly hatched second-stage juveniles (J2) were collected and stored (Goodey, 1957). All experiments were carried out with J2 obtained within 72 h.

### Laboratory experiments

#### Mortality

Four concentrations (125, 250, 500, and 1,000 μg/mL) were prepared in 0.3% Tween 20 for each compound. There were four replicates of each concentration, each treatment comprising about 100 of *M. incognita* J2. The bioassay was performed in cavity watch glasses with four batches of 25 J2 individually in 2 ml of each test solution. Distilled water with 0.3% tween 20 was used as control. Treatments were incubated at 25 ± 2°C and nematode mortality was assessed after 72 h of exposure. According to the methods of Finney (1971) and Cheng et al. (2015), the lethal concentrations for half of the treated J2 (LC_50_) for each treatment were determined.

#### Hatching

Nearly 100 eggs were selected from mature egg masses using sterile forceps from roots free of soil and were transferred to glass bottles with 2 mL of each concentration (125, 250, 500, and 1,000 μg/ml) of carvone, cuminaldehyde, cineole, and linalool which were prepared in distilled water. Four replicates were made in each treatment. The hatched juveniles were counted under a stereo microscope (Commack, NY, United States) after incubation for 7 days at room temperature (25 ± 2°C). The rate of hatching inhibition was calculated, and Probit analysis was used to measure the inhibition concentration for half of eggs hatching (IC_50_) values (Finney, 1971; Damascena et al., 2019).

### Greenhouse experiment

The efficacy of the compounds on *M. incognita* reproduction under greenhouses was also evaluated at a concentration of 250 mg/kg soil at temperature ranged between 25 and 30°C. Oxamyl was used at a concentration of 8 mL/L as a reference nematicide. Forty-day-old seedlings were planted singly in a plastic container (25 cm in diameter) filled with 2 kg sandy clay soil (3:1, v: v, sand: clay) sterilized with steam. One week after planting and selecting the seedlings with the best growth of the roots, 100 mL of each compound solution was injected into the soil around the stem of the plant (soil drenched). After 2 days, each pot was inoculated with an initial inoculum level (500 juveniles in 100 mL per pot) from root-knot nematodes around the stem of the plant within a radius of 2 cm. After 2 days, each pot was inoculated with 500 J2 around the stem of each plant within a radius of 2 cm. After 10 days, plants were treated again with the compounds as mentioned above. There were four replicates for each treatment, including untreated controls. Two months after nematode inoculation, tomato plants were carefully uprooted and cleaned under running tap water.

Plant height and fresh and dry weight of the plant were recorded. In addition, the number of galls and measurements related to nematodes were recorded, including the gall index, egg masses, and the final population density (number of J2) (Barker, 1985). Egg masses number was determined according to Daykin and Hussey (1985) method by dipping the roots in a 0.015% Phloxine-B coloring solution for 20 min. The quantity of J2 per 250 cm^3^ soil was measured using serial sieves and an adapted Baermann’s technique on a slide under a stereomicroscope (Goodey, 1957). Roots are indexed on a scale as described by Barker (1985) from 0 to 5 scale (0; 0–10%, 1; 11–20%, 21–50%, 3; 51–80%, 4; 81–90%, and 5; 91–100%). The reduction percentage (R%) of nematode parameters were calculated according to the following equation:


$$\begin{array}{c} \mathrm{ReductionPercentage}\left(R\%\right)\\ \;=\left(\mathrm{Population}\mathrm{of}\mathrm{control}-\mathrm{Population}\mathrm{of}\mathrm{treatment}\right)/\\ \;\mathrm{Population}\mathrm{of}\mathrm{control}\times 100 \end{array}$$

### Analysis of defense related genes expression

Tomato plants were treated with the selected monoterpenes and inoculated with *M. incognita* as described previously. RNA extraction was carried out at 2 days after nematode inoculation from tomato leaves using RNA Purification Kit (Thermo Scientific, Fermentas, #K0731). Complementary DNA (cDNA) was synthesized using Reverse Transcription Kits (Thermo Scientific, Fermentas, #EP0451). Quantitative RT-PCR (qRT-PCR) with SYBR Green was utilized to measure the expression of the target genes (*PR1* and *PAL*), with LeUBI3 Table 1 as an internal reference following the manufacturer protocol (Thermo Scientific, United States, # K0221). The 2^–ΔΔCt^ method was used to normalize the numbers of target genes’ critical thresholds (Ct) with the numbers (Ct) of a housekeeping gene (*LeUBI3*) (Livak and Schmittgen, 2001).

**TABLE 1:** Forward and reverse primers.

Gene	Forward primer	Reverse primer	Size	Accession number	References
*PR1*	GCCAAGCTATAACTACGCTACCAAC	GCAAGAAATGAACCACCATCC	139	DQ159948	Safaie-Farahani and Taghavi, 2017
*PAL*	CTGGGGAAGCTTTTCAGAATC	TGCTGCAAGTTACAAATCCAGAG	150	AW035278	Safaie-Farahani and Taghavi, 2017
*LeUBI3*	TCCATCTCGTGCTCCGTCT	GAACCTTTCCAGTGTCATCAACC	144	X58253	Song et al., 2015

### Field experiment

This experiment was performed to evaluate the effectiveness of carvone, cineole, cuminaldehyde, and linalool under field conditions compared to oxamyl. Experiments were conducted in fields naturally infested with *M. incognita* in Baltim, Kafr Elsheikh Governorate. All treatments were arranged in a split plot design with four replications. Each treatment consisted of one row and each row was 1 meter wide. The general cultivation recommendations for tomato plants were applied during the 2019 and 2020 growing seasons. Untreated plants were used as a control. After 2 months of treatment, the tomato roots were uprooted and washed. Then the numbers of nematodes were calculated as previously described in addition to determining the tomato yield.

### Statistical analysis

To determine LC_50_ and IC_50_, mortality and inhibition rates were subjected to a probit analysis (Finney, 1971). If the 95 percent confidence limits did not overlap, the LC_50_ and IC_50_ values were considered significantly different. All experiments were repeated three times. Greenhouse and field data were statistically analyzed using ANOVA. Averages were compared by Fisher’s LSD test. The analysis was performed using XLSTAT PRO (statistical analysis software, Addinsoft).

## Results

### Efficacy of monoterpenes on the second-stage juveniles and eggs hatchability

The efficiency of the selected compounds on the J2 of *M. incognita* was evaluated under laboratory conditions Table 2. The results indicated that the highest mortality in the number of J2 was recorded for carvone followed by the cuminaldhyde, linalool, and cineole, where the LC_50_ values were 123.5, 172.2, 354.9, 466.4, and 952.3 μg/mL, respectively.

**TABLE 2:** Effect of the used compounds on the mortality.

Treatment	LC_50_ (μg/mL)	95% Confidence Limits (μg/ml)		Slope ± S.E
		Lower	Upper	
Cineole	952.3a	763.1	1277.4	1.84 ± 0.24
Cuminaldehyde	172.2 c	145.1	199.9	2.35 ± 0.26
Linalool	354.9b	301.3	439.4	1.53 ± 0.18
Carvone	123.5 d	116.7	164.2	2.77 ± 0.27

Different letters mean 95 % confidence limits did not overlap and the LC_50_ values were significantly different.

The efficacy of the selected compounds on hatching inhibition of *M. incognita* eggs under laboratory conditions was presented in Table 3. The results showed that the carvone was the most effective compound on hatching inhibition, followed by cuminaldhyde, cineole, and linalool, where the IC_50_ values were 88.2, 102.1, 480, 646.9, and 780.4 μg/mL, respectively.

**TABLE 3:** Effect of the used compounds on the hatching.

Treatment	LC_50_ (μg/mL)	95% Confidence Limits		Slope ± S.E
		Lower	Upper	
Cineole	646.9b	535.7	848.3	1.56 ± 0.19
Cuminaldehyde	102.1 c	81.9	120.1	3.23 ± 0.29
Linalool	780.4 a	609.1	1144.1	1.35 ± 0.18
Carvone	88.2 c	66.9	109.3	2.43 ± 0.28

Different letters mean 95 percent confidence limits did not overlap and the LC_50_ values were significantly different.

### Effect of the selected monoterpenes on growth characteristics of tomato

The effect of the selected compounds on certain growth characteristics of tomato crop (plant height as well as fresh and dry weight of the plant) under greenhouse conditions compared to the recommended nematicide was presented in Table 4. The measured growth characters were improved in tomato plants treated with the selected monoterpenes compared to untreated control. The results showed that the measured growth characters (plant height as well as fresh and dry weight of the plant) were the highest in tomato plants treated with carvone followed by the cuminaldhyde, linalool, nematicide, and cineole, respectively.

**TABLE 4:** Effect of treatments on the plant height (cm), shoot fresh and dry weights (g).

Treatments	Plant height	Shoot weight	
		Fresh weight	Dry weight
Control (healthy)	47.5 ± 1.25^a^	178.15 ± 3.25^a^	20.7 ± 0.12^a^
Control (infested)	27.4 ± 0.75^e^	64.2 ± 1.17^e^	7.2 ± 0.11e
Cineole	41.1 ± 1.10^d^	133.7 ± 2.65^d^	15.1 ± 0.45^d^
Cuminaldehyde	44.8 ± 1.12^b^	165.9 ± 1.85^b^	18.3 ± 0.36^b^
Linalool	42.7 ± 1.45^c^	151.1 ± 1.36^c^	17.1 ± 0.38^c^
Carvone	45.9 ± 2.10^b^	167.2 ± 2.47^b^	18.4 ± 0.37^b^
Oxamyl	43.3 ± 0.95^c^	149.4 ± 1.10^c^	16.9 ± 0.74^c^

Weight of tomato plants grown in soil infested with nematode under greenhouse conditions.

Statistical comparisons were made among treatments within a single column.

The different letters represent significant differences using Fisher’s LSD test at *P* ≤ 0.05.

Each mean value came from four replicates.

### Efficiency of the selected monoterpenes on root knot nematodes of tomato under greenhouse conditions

**FIGURE 1:**
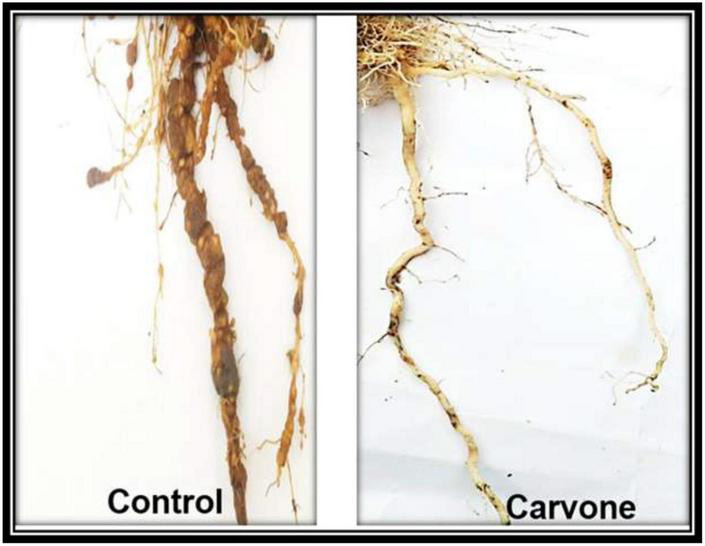
Symptoms on tomato roots treated with carvone and non-treated control plants.

The results in Table 5 showed that the recommended nematicide and the carvone were the most effective treatments at the level of all the measurements taken (root-galling, egg masses, and the number of J2 per 250 cm^3^ soil), followed by cuminaldhyde, linalool, and cineole, respectively. Figure 1 shows a comparison between symptoms of RKN attack observed on tomato roots treated with carvone compared to control (untreated) one.

**TABLE 5:** Effect of treatments on root galling, egg masses, and juveniles in tomato plants under greenhouse conditions.

Treatments	Root-galling		Egg-masses		Nematode population	
	Gall index	Reduction (%)	Number of egg-masses/Plant	Reduction (%)	No. of J2/250 cm^3^ soil	Reduction (%)
Control healthy	0.0	–	0.0	–	0.0	–
Control infested	4.8 ± 0.13^a^	–	147.7 ± 2.58^a^	–	1876 ± 3.10^a^	–
Cineole	3.0 ± 0.10^b^	37.5 ± 1.10	78.3 ± 1.34^b^	47.0 ± 0.84	1229 ± 2.65^b^	34.5 ± 1.12
Cuminaldehyde	1.5 ± 0.07^d^	68.8 ± 1.34	34.8 ± 1.97^d^	76.4 ± 1.25	629 ± 2.97^d^	66.5 ± 0.74
Linalool	2.0 ± 0.10^c^	52.1 ± 1.17	51.2 ± 1.10^c^	65.3 ± 0.77	801 ± 4.10^c^	57.3 ± 0.69
Carvone	1.2 ± 0.06^e^	75.0 ± 1.28	23.1 ± 1.11^e^	84.4 ± 0.89	513 ± 2.74^e^	72.7 ± 1.14
Oxamyl	1.1 ± 0.10^e^	77.1 ± 1.55	18.7 ± 0.89^f^	87.3 ± 1.10	491 ± 3.14^f^	73.8 ± 1.34

Statistical comparisons were made among treatments within a single column.

The different letters represent significant differences using Fisher’s LSD test at P ≤ 0.05.

Each mean value came from four replicates.

### Efficiency of selected monoterpenes on root knot nematodes of tomato under field conditions

The results in Table 6 showed that the recommended nematicide and the carvone were the most effective treatments with respect to all measurements taken (root-galling, the number of J2 in 250 cm^3^ soil, and yield of tomato as a ton per hectare), followed by cuminaldhyde, linalool and cineole, respectively.

**TABLE 6:** Effect of treatments on root galling, nematode population and yield in tomato plants grown in soil infested with *M. incognita* under field conditions.

Treatments	Root-galling		Nematode population		Yield (Ton/Hectare)
	Gall index	Reduction (%)	No. of J2/250 cm^3^ soil	Reduction (%)	
Control infested	4.2 ± 0.16^a^	–	2611 ± 1.45^a^	–	15.3 ± 0.25^e^
Cineole	3.3 ± 0.14^b^	21.4 ± 0.74	1530 ± 2.84^b^	41.4 ± 2.10	25.7 ± 0.23^d^
Cuminaldehyde	1.9 ± 0.10^d^	54.8 ± 1.10	1130 ± 2.64^d^	56.7 ± 1.57	31.6 ± 0.54^b^
Linalool	2.6 ± 0.12^c^	38.1 ± 0.37	1258 ± 3.97^c^	51.8 ± 1.97	27.5 ± 0.78^c^
Carvone	1.8 ± 0.10^d^	57.1 ± 1.16	1102 ± 1.79^d^	57.8 ± 1.35	33.7 ± 0.69^a^
Oxamyl	1.4 ± 0.10^e^	66.7 ± 1.74	1031 ± 2.67^e^	60.5 ± 1.68	30.4 ± 0.47^b^

Statistical comparisons were made among treatments within a single column.

Superscript of different letters represents significant differences using Fisher’s LSD test at P ≤ 0.05.

Each mean value came from five replicates.

### Expression of defense-related genes

**FIGURE 2:**
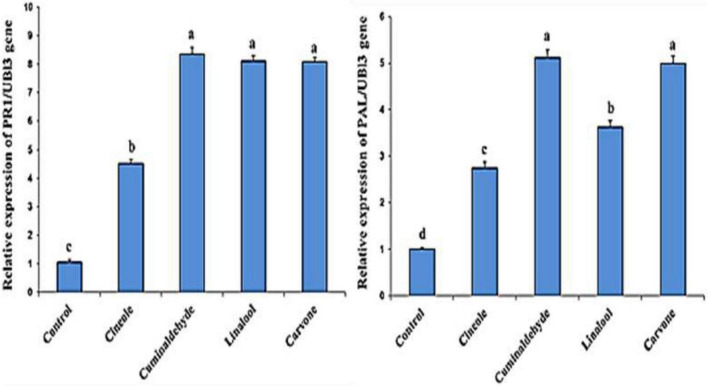
Effect of different treatments on the expression levels of *PR1* and *PAL* genes. Columns represent mean values. The control corresponds to inoculated and non-treated plants. Bars indicate standard errors. Different letters above columns indicate significant differences by Fisher’s LSD test at significant values of *P* ≤ *0.5*.

The effect of the compounds on the expression of defense genes (*PR1* and *PAL* gens) in the treated and inoculated tomato plants is investigated Figure 2. Higher transcription levels of *PR1* and *PAL* gens in treated tomato plants in comparison to untreated control was obtained. The findings also showed that the highest level of expression in tomato plants was cuminaldhyde followed by carvone, linalool, and cineole, respectively.

## Discussion

*Meloidogyne incognita* causes root-knot, which is an important disease in different regions of the world (Onyeke and Akueshi, 2012; Devi and Kumari, 2014). *M. incognita* has a wide range of host plants, causing yield losses in severely infested fields (Onyeke and Akueshi, 2012; Mukhtar et al., 2014). In this study, under laboratory, greenhouse, and field conditions, the four monoterpenes (carvone, cuminaldehyde, cineole, and linalool) demonstrated nematicidal behavior against the RKN. Monoterpenes are the primary components of aromatic plant essential oils, and they are responsible for the majority of the biological activities of plant extracts (Sacchetti et al., 2005; Bakkali et al., 2008; Abdel Rasoul, 2013; Naz et al., 2013; Khan et al., 2020). Our results are in line with many investigation which reported that several essential oils and some of their major components have nematicidal activity against RKN (Abdel Rasoul, 2013; Naz et al., 2013; Lu et al., 2017; D’Addabbo et al., 2021). The most effective compounds against *M. incognita* in this study were carvone and cuminaldehyde, which is in line with the findings of Abdel Rasoul (2013). The high efficacy of carvone and cuminaldehyde against RKN may be due to the presence of a hydroxyl or carbonyl group in these two compounds. This indicates that the functional group is very important in their nematicide activity (Echeverrigaray et al., 2010).

Carvone, cominaldehyde, cineole, and linalool were evaluated in controlling *M. incognita* in tomato plants under greenhouse and field conditions. The results obtained in this study showed that they significantly reduced the numbers of galls, egg masses, and J2 in the root system compared to the infected control. These results were similar to those reported by other studies on essential oils (Echeverrigaray et al., 2010; Abdel Rasoul, 2013). The results of this study, however, differ from those of others in that the monoterpenes utilized were examined at all levels in the laboratory, greenhouse, and field. The ability of tomato plants to display induced resistance to RKN was also investigated by looking at the expression of defense genes in treated tomato plants.

Several studies have demonstrated the nematicidal activity of plant extracts in several plant species (Barbosa et al., 2010; Sivakumar and Gunasekaran, 2011; Zahradnikova and Petrikova, 2013). For example, the severity of *M. incognita* was significantly reduced in plants treated with neem extracts (Sivakumar and Gunasekaran, 2011). Pumpkin oil also showed nematicidal activity (Ayaz et al., 2015). Moreover, the treatment with watercress oil resulted in a significant decrease in symptoms caused by *M. hapla* nematode disease and a significant increase in the fruit yield (Zahradnikova and Petrikova, 2013).

A plant self-defense mechanism against infectious parasites can be initiated within plant root consisting of many biologically active secondary metabolites. There are a limited number of researches conducted to understand the effectiveness of induced resistance within the same species (Navyashree et al., 2021). Different studies have indicated the use of plant resistance inducers to alleviate these adversities, increase plant metabolic activity, and develop a defense mechanism against various parasites. This kind of studies will also add value to our understanding of naturally occurring interactions between plant and RKN (Dutta et al., 2015).

One of the primary impacts of changes in the gene expression of plants is to elicit resistance to a large variety of pathogens and parasites including nematodes (Druzhinina et al., 2011; Cameron et al., 2013). In this study, we analyzed changes in the expression of defensive genes after treating tomato plants (soil drench) with control measures. The defensive genes analyzed in this study were highly expressed in tomato plants treated with the tested inducers. *PR1* and *PAL* genes were overexpressed after treatment with the selected compounds. This is in line with Sharaf et al. (2016) results, who reported that the expression of defense-related genes elevated in nematode-infected tomato plants and treated with inducers.

Plant extracts and plant residues, alone or in addition to physical measures, have been proven promising effects in weeds, microbial, and insect control, and certain commercial natural oils are currently accessible for organic agriculture purposes (Dayan et al., 2009). Essential oils and organic amendments derived from essential oil-rich plants have been used to successfully protect plants against phytonematodes (Pérez et al., 2003; Onifade, 2007). For the mode of action of essential oils, it has been found that some essential oils have genotoxic activity in *Drosophila melanogaster* (Karpouhtsis et al., 1998; Enan, 2001; Lazutka et al., 2001), to activate octopamine receptors (Karpouhtsis et al., 1998; Enan, 2001), and interfering with GABA receptors for insects (Priestley et al., 2003). As lipophiles, essential oils and terpenoids interfere with the cytoplasmic membrane of yeasts, destroying the structure of carbohydrates, fatty acids, and phospholipids, causing mitochondrial membrane depolarization and leakage of radicals, cytochrome C, calcium ions, and proteins (Bakkali et al., 2008). The presence of phenols, aldehydes, and alcohols in essential oils has been related to their *in vitro* cytotoxic activity (Bruni et al., 2004; Onifade et al., 2008).

The results showed that the plants infected with nematodes and non-treated with selected compounds displayed a delay in growth as plant height decreased, and the fresh and dry root weights were significantly reduced due to nematode infection, which is in line with Radwan et al. (2009) and Elsayed and Edrees (2014). The significant increase in growth characteristics is the primary criterion for judging the incidence of induced systemic resistance (ISR) in nematode-infected plants treated with inducible control agents (Sharaf et al., 2016). The results in this study clearly indicated a significant improvement in the growth characteristics and productivity of tomato plants treated with the selected monoterpenes. This improvement may result in the ability of the treatments to reduce nematode infection on the roots. This is because healthy or low-infected roots have the ability to transport water and nutrients from the soil through the xylem, which is reflected in the growth of tomato plants (Bakr and Hewedy, 2018).

## Conclusion

The present results showed that the used monoterpenes as plant-derived natural compounds possess strong nematicidal activity against *M. incognita* under laboratory, greenhouse, and field conditions. Antihatching and/or anti-juveniles action against *M. incognita* was revealed in most tested monoterpenoids. For the management of *M. incognita*, carvone proved to be the most effective natural nematicide, as shown by its ability to reduce egg hatching and the number of J2. The growth and yield characters of tomato treated with the tested compounds increased compared to untreated control. Since phytochemicals have high nematicidal ability, the current study suggests that they may be used as alternatives in an integrated disease management program against *M. incognita.*

## Data availability statement

The raw data supporting the conclusions of this article will be made available by the authors, without undue reservation.

## Author contributions

AD and ME: conceptualization, formal analysis, investigation, data curation, writing – original draft preparation, and supervision. ME: methodology and software. AD, AA, SB, MS, MK, and ME: validation. SB, AA, AA-A, MK, and ME: resources. AD, AA, SB, and ME: writing – review and editing. SB, AA, AA-A, and ME: visualization and funding acquisition. AD, AA-A, and ME: project administration. All authors have read and agreed to the published version of the manuscript.

## References

[B1] Abdel Rasoul M. A. (2013). Evaluation of nematicidal effects of monoterpenes against root-knot nematode, *Meloidogyne incognita*. *J. Plant Protect. Pathol. Mansoura Univ.* 4 445–456.

[B2] Abd-Elgawad M. M. M. (2014). Yield losses by phytonematodes: Challenges and opportunities with special reference to Egypt. *Egyp. J. Agronematol.* 13 75–94. 10.21608/ejaj.2014.63633

[B3] Abd-Elgawad M. M. M. (2020). Optimizing biological control agents for controlling nematodes of tomato in Egypt. *Egypt. J. Biol. Pest Cont.* 30:58. 10.1186/s41938-020-00252-x

[B4] Ayaz E. Gökbulut C. Coşkun H. Türker A. Özsoy Ş Ceylan K. (2015). Evaluation of the anthelmintic activity of pumpkin seeds (*Cucurbita maxima*) in mice naturally infected with *Aspiculuris tetraptera*. *J. Pharm. Phyt.* 7 189–193. 10.5897/JPP2015.0341

[B5] Bakkali F. Averbeck S. Averbeck D. Idaomar M. (2008). Biological effects of essential oils—A review. *Food Chem. Toxicol.* 46 446–475. 10.1016/j.fct.2007.09.106 17996351

[B6] Bakr R. A. Hewedy O. M. (2018). Monitoring of systemic resistance induction in tomato against *Meloidogyne incognita*. *J. Plant Pathol. Microbiol.* 9 2–5. 10.4172/2157-7471.1000464

[B7] Barbosa P. Lima A. S. Vieira P. Dias L. S. Tinoco M. T. Barroso J. G. (2010). Nematicidal activity of EOs and volatiles derived from portuguese aromatic flora against the pinewood nematode, *Bursaphelenchus xylophilus*. *J. Nematol.* 42 8–16. 22736831 PMC3380513

[B8] Barker K. R. (1985). “Nematode extraction and bioassay,” in *An advanced treatise on meloidogyne, methodology.* eds Barker K. R. Carter C. C. Sasser J. N. (Raleigh, NC: North Carolina State University Graphics), 19–35.

[B9] Bruni R. Médici A. Andreotti E. Fantin C. Muzzoli M. Dehesa M. (2004). Chemical composition and biological activities of Ishpingo essential oil, a traditional Ecuadorian spice from *Ocotea quixos* (Lam.) Kosterm. (Lauraceae) flower calices. *Food Chem.* 85 415–421. 10.1016/j.foodchem.2003.07.019

[B10] Caillaud M. C. Dubreuil G. Quentin M. Perfus- Barbeoch L. Lecomte P. Engler J. D. A. (2008). Root-knot nematodes manipulate plant cell functions during a compatible interaction. *J. Plant Physiol.* 165 104–113. 10.1016/j.jplph.2007.05.007 17681399

[B11] Cameron D. D. Neal A. L. Van-Wees S. C. M. Ton J. (2013). Mycorrhiza-induced resistance: More than the sum of its parts? *Trends Plant Sci.* 18 539–545. 10.1016/j.tplants.2013.06.004 23871659 PMC4194313

[B12] Campos ÂD. Ferreira A. G. Hampe M. M. V. Antunes I. F. Brancão N. Silveira E. P. (2003). Induction of chalcone synthase and phenylalanine ammonia-lyase by salicylic acid and *Colletotrichum lindemuthianum* in common bean. *Braz. J. Plant Physiol.* 15 129–134. 10.1590/S1677-04202003000300001

[B13] Cheng X. Liu X. Wang H. Ji X. Wang K. Wei M. (2015). Effect of emamectin benzoate on root-Knot nematodes and tomato yield. *PLoS One* 10:e0141235. 10.1371/journal.pone.0141235 26509680 PMC4624971

[B14] D’Addabbo T. Laquale S. Argentieri M. P. Bellardi M. G. Avato P. (2021). Nematicidal activity of essential oil from Lavandin (*Lavandula intermedia* Emeric ex Loisel.) as related to chemical profile. *Molecules* 26:6448. 10.3390/molecules26216448 34770856 PMC8587996

[B15] Damascena A. P. Ferreira J. C. A. Costa M. G. S. de Araujo Junior L. M. Wilcken S. R. S. (2019). Hatching and mortality of *Meloidogyne enterolobii* under the interference of entomopathogenic nematodes in vitro. *J. Nematol.* 17 e2019–e2058. 10.21307/jofnem-2019-058 34179792 PMC6909014

[B16] Dayan F. E. Cantrell C. L. Duke S. O. (2009). Natural products in crop protection. *Biorg. Med. Chem.* 17 4022–4034. 10.1016/j.bmc.2009.01.046 19216080

[B17] Daykin M. Hussey R. (1985). “Staining and histopathological techniques in nematology,” in *An advanced treatise on Meloidogyne. Method*, Vol. 2 eds Barker K. R. Carter C. C. Sasser J. N. (Raleigh, N.C: Department of Plant Pathology, North Carolina State University), 39–48.

[B18] Devi M. L. Kumari N. V. (2014). Prevalence of *Meloidogyne* species in different crops of Indian sub-continent: A review. *Inter. J. Adv. Res.* 2 530–537.

[B19] Druzhinina I. S. Seidl-Seiboth V. Herrera-Estrella A. Horwitz B. A. Nenerley C. M. Monte E. (2011). Trichoderma: The genomics of opportunistic success. *Nat. Rev.* 9 749–759. 10.1038/nrmicro2637 21921934

[B20] Dutta A. Mandal A. Kundu A. Malik M. Chaudhary A. Khan M. R. (2021). Deciphering the behavioral response of *Meloidogyne incognita* and *Fusarium oxysporum* toward mustard essential oil. *Front. Plant Sci.* 26:1791. 10.3389/fpls.2021.714730 34512695 PMC8427441

[B21] Dutta K. T. Papolu K. P. Banakar P. Choudhary D. Sirohi A. Rao U. (2015). Tomato transgenic plants expressing hairpin construct of a nematode protease gene conferred enhanced resistance to root-knot nematodes. *Front. Microbiol.* 6:260. 10.3389/fmicb.2015.00260 25883594 PMC4381642

[B22] Echeverrigaray S. Zacaria J. Beltrão R. (2010). Nematicidal activity of monoterpenoids against the root-knot nematode *Meloidogyne incognita* Sergio. *Nematology* 100 199–203. 10.1094/PHYTO-100-2-0199 20055654

[B23] Elsayed I. A. Edrees O. N. (2014). Potency evaluation of *Pseudomonas* strains against root- knot nematode infecting tomato. *Inter. J. Adv. Res.* 28 602–608. 10.13140/RG.2.1.4708.1124

[B24] El-Shafeey I. E. El-Khateeb N. M. M. Elsharkawy M. M. Elsary G. S. Homayed S. H. (2019). Induction of systemic resistance against *Meloidogyne incognita* by different chemical and biological inducers in tomato plants. *Fres. Environ. Bull.* 28 6692–6700.

[B25] Enan E. (2001). Insecticidal activity of essential oils: Octopaminergic sites of action. *Comp. Biochem. Physiol. C Toxicol. Pharmacol.* 130 325–337. 10.1016/S1532-0456(01)00255-1 11701389

[B26] Farahani A. S. Taghavi S. M. Afsharifar A. Niazi A. (2016). Changes in expression of pathogenesis-related gene 1, pathogenesis-related gene 2, phenylalanine ammonia-lyase and catalase in tomato in response to *Pectobacterium carotovorum* subsp carotovorum. *J. Plant Pathol.* 98 525–530. 10.4454/JPP.V98I3.036 32896216

[B27] Finney J. C. (1971). *Probit analysis.* London: Cambridge University Press, 33.

[B28] Ghahremani Z. Escudero N. Saus E. Gabaldón T. Sorribas F. J. (2019). *Pochonia chlamydosporia* induces plant-dependent systemic resistance to *Meloidogyne incognita*. *Front. Plant Sci.* 10:945. 10.3389/fpls.2019.00945 PMC6700505 31456811

[B29] Goodey J. B. (1957). *Laboratory methods for work with plant and soil nematodes.* London: Ministry of Agriculture, Fisheries and Food, 47.

[B30] Hussey R. S. Barker K. R. (1973). A comparison of methods of collecting *Meloidogyne* spp. including a new technique. *Plant Dis. Rep.* 57 1925–1928.

[B31] Karpouhtsis R. Pardali E. Feggou E. Kokkini S. Scouras Z. G. Mavragani-Tsipidou P. (1998). Insecticidal and genotoxic activities of oregano essential oils. *J. Agric. Food Chem.* 46 1111–1115. 10.1021/jf970822o

[B32] Keerthiraj M. Mandal A. Dutta T. K. Saha S. Dutta A. Singh A. (2021). Nematicidal and molecular docking investigation of essential oils from *Pogostemon cablin* ecotypes against *Meloidogyne incognita*. *Chem. Biodivers.* 18:e2100320. 10.1002/cbdv.202100320 34245651

[B33] Kessel C. (2003). Fertilizing tomatoes. *Veg. Prod. Pub.* 363 11–27.

[B34] Khan R. Naza I. Hussain S. Khan R. A. A. Ullah S. Rashid M. U. (2020). Phytochemical management of root knot nematode (*Meloidogyne incognita*) kofoid and white chitwood by *Artemisia* spp. in tomato (*Lycopersicon esculentum* L.). *Braz. J. Biol.* 80 829–838. 10.1590/1519-6984.222040 31800766

[B35] Kundu A. Dutta A. Mandal A. Negi L. Malik M. Puramchatwad R. (2021). A comprehensive in vitro and in silico analysis of nematicidal action of essential oils. *Front. Plant Sci.* 11:614143. 10.3389/fpls.2020.614143 33488658 PMC7820373

[B36] Kundu A. Saha S. Walia S. Dutta T. K. (2016). Antinemic potentiality of chemical constituents of *Eupatorium adenophorum* Spreng leaves against *Meloidogyne incognita*. *Natl. Acad. Sci. Lett.* 39 145–149.

[B37] Lazutka J. R. Mierauskien J. Slap G. Dedonyt V. (2001). Genotoxicity of dill (*Anethum graveolens* L.), peppermint (*Mentha piperita* L.) and pine (*Pinus sylvestris* L.) essential oils in human lymphocytes and *Drosophila melanogaster*. *Food Chem. Toxicol.* 39 485–492. 10.1016/s0278-6915(00)00157-5 11313115

[B38] Livak K. J. Schmittgen T. D. (2001). Analysis of relative gene expression data using real-time quantitative PCR and the 2-ΔΔCT method. *Methods* 25 402–408. 10.1006/meth.2001.1262 11846609

[B39] Lu H. Xu S. Zhang W. Xu C. Li B. Zhang D. (2017). Nematicidal activity of trans-2-Hexenal against southern root-knot nematode (*Meloidogyne incognita*) on tomato plants. *J. Agric. Food Chem.* 65 544–550. 10.1021/acs.jafc.6b04091 28048941

[B40] Lutuf H. Nyaku S. T. Cornelius E. W. Yahaya S. A. J. Acheampong M. A. (2018). Prevalence of plant-parasitic nematodes associated with tomatoes in three agro-ecological zones of Ghana. *Ghana. J. Agric. Sci.* 52 83–94.

[B41] Malash N. Flowers T. J. Ragab R. (2005). Effect of irrigation systems and water management practices using saline and non-saline water on tomato production. *Agric. Wat. Manag.* 78 25–38.

[B42] Mauch-mani B. Slusarenko A. J. (1996). Production of salicylic acid precursors is a major function of phenylalanine ammonia-lyase in the resistance of *Arabidopsis* to *Peronospora parasitica*. *Plant Cell* 8 203–212. 10.1105/tpc.8.2.203.1007/s00438-008-0322-9 12239383 PMC161092

[B43] Mazarei M. Liu W. Al-Ahmad H. Arelli P. R. Pantalone V. R. Stewart C. N. Jr. (2011). Gene expression profiling of resistant and susceptible soybean lines infected with soybean cyst nematode. *Theor. Appl. Genet.* 123 1193–1206. 10.1007/s00122-011-1659-8 21800143

[B44] Mishina T. E. Zeier J. (2007). Bacterial non-host resistance: Interactions of *Arabidopsis* with non-adapted *Pseudomonas* syringae strains. *Physiol. Plant* 131 448–461. 10.1111/j.1399-3054.2007.00977.x 18251883

[B45] Mitsuhara I. Iwai T. Seo S. Yanagawa Y. Kawahigasi H. Hirose S. (2008). Characteristic expression of twelve rice PR1 family genes in response to pathogen infection, wounding, and defense-related signal compounds. *Mol. Genet. Genomics* 279 415–427. 10.1007/s00438-008-0322-9 18247056 PMC2270915

[B46] Molinari S. (2011). Natural genetic and induced plant resistance, as a control strategy to plant-parasitic nematodes alternative to pesticides. *Plant Cell Rep.* 30 311–323. 10.1007/s00299-010-0972-z 21184231

[B47] Mukhtar T. Hussain M. A. Kayani M. Z. Aslam M. N. (2014). Evaluation of resistance to root-knot nematodes (*Meloidogyne incognita*) in okra cultivars. *Crop Protect.* 56 25–30. 10.1016/j.cropro.2013.10.019

[B48] Navyashree B. Dharmashekar C. Shivamallu C. Balasubramanian S. Prasad S. K. Prasad K. S. (2021). Plant induced resistance in *Solanacearum lycopersicum* species against root knot nematodes. *J. App. Biol. Biotech.* 9, 88–95. 10.7324/JABB.2021.9112

[B49] Naz I. Palomares-Rius J. E. Saifullah, Blok V. Khan M. R. Ali S. (2013). In vitro and in planta nematicidal activity of *Fumaria parviflora* (Fumariaceae) against the southern root knot nematode *Meloidogyne incognita*. *Plant Pathol.* 62 943–952. 10.1111/j.1365-3059.2012.02682.x

[B50] Ogwulumba S. I. Ogwulumba I. C. (2018). Screen house management of *Meloidogyne javanica* (Treub) in UC82B tomato (*Solanum lycopersicum*) with leaf extract of *Jatropha curcas*. *J. Entomol. Nematol.* 10 33–36. 10.5897/JEN2017.0168

[B51] Oka Y. Koltai H. Bar-Eyal M. Mor M. Sharon E. Chet I. (2000). New strategies for the control of plant-parasitic nematodes. *Pest Manag. Sci.* 56 983–988.

[B52] Onifade A. K. (2007). Effect of essential oils from five *Ocimum* sp. on the pathogenicity of *Pratylenchus brachyurus* (Godfrey) in tomato. *Agric. J.* 2 185–191.

[B53] Onifade A. K. Fatope M. O. Deadman M. L. Al-Kindy S. M. Z. (2008). Nematicidal activity of *Haplophyllum tuberculatum* and *Plectranthus cylindraceus* oils against *Meloidogyne javanica*. *Biochem. Syst. Ecol.* 36 679–683. 10.1016/j.bse.2008.05.005

[B54] Onyeke C. C. Akueshi C. O. (2012). Pathogenicity and reproduction of *Meloidogyne incognita* (Kofoid and White) chitwood on African yam bean, *Sphenostylis stenocarpa* (Hochst Ex. A. Rich) Harms accessions. *African J. Biotechnol.* 11 1607–1616. 10.5897/AJB11.3000

[B55] Pérez M. P. Navas-Cortés J. A. Pascual-Villalobos M. J. Castillo P. (2003). Nematicital activity of essential oils and organic amendments from Asteraceae against root-knot nematodes. *Plant Pathol.* 52 395–401. 10.1046/j.1365-3059.2003.00859.x

[B56] Priestley C. M. Williamson E. M. Wafford K. A. Sattelle D. B. (2003). Thymol, a constituent of thyme essential oil, is a positive allosteric modulator of human GABAA receptors and a homo-oligomeric GABA receptor from *Drosophila melanogaster*. *Brit. J. Pharmacol.* 140 1363–1372. 10.1038/sj 14623762 PMC1574153

[B57] Radwan M. A. El-Maadawy E. K. Kassem S. I. Abu-Elamayem M. M. (2009). Oil cakes soil amendment effects on *Meloidogyne incognita* infecting tomato. *Arch. Phytopathol. Plant Protect.* 42 58–64. 10.1080/03235400600940830

[B58] Sacchetti G. Maietti S. Muzzoli M. Scaglianti M. Manfredini S. Radice M. (2005). Comparative evaluation of 11 essential oils of different origin as functional antioxidants, antiradicals and antimicrobials in food. *Food Chem.* 91 621–632. 10.1016/j.foodchem.2004.06.031

[B59] Safaie-Farahani A. Taghavi S. M. (2017). Transcript analysis of some defense genes of tomato in response to host and non-host bacterial pathogens. *Mol. Biol. Res. Commun.* 6 177–183. 10.22099/mbrc.2017.25600.1273 29383321 PMC5762990

[B60] Sharaf A. M. A. Kailla A. M. Attia A. S. Nofal M. M. (2016). Induced resistance in tomato plants against root knot nematode using biotic and abiotic inducers. *Int. J Adv. Res. Biol. Sci.* 3 31–46. 10.22192/ijarbs.2016.03.11.004

[B61] Sivakumar M. Gunasekaran K. (2011). Management of root-knot nematodes in tomato, chilli and brinjal by neem oil formulations. *J. Biopest.* 4 198–200.

[B62] Song Y. Chen D. Lu K. Sun Z. Zeng R. (2015). Enhanced tomato disease resistance primed by arbuscular mycorrhizal fungus. *Front. Plant Sci.* 6:786. 10.3389/fpls.2015.00786 26442091 PMC4585261

[B63] USDA (2005). *USDA nutrient database for standard reference, Release 18.* Washington, D.C: U.S. Dept. of Agriculture, Agricultural Research Service.

[B64] Vallad G. E. Goodman R. M. (2004). Systemic acquired resistance and induced systemic resistance in conventional agriculture. *Crop Sci.* 44 1920–1934. 10.2135/cropsci2004.1920

[B65] Van Loon L. C. Bakker P. A. H. M. Pieterse C. M. J. (1998). Systemic resistance induced by rhizosphere bacteria. *Ann. Rev. Phytopathol.* 36 453–483. 10.1146/annurev.phyto.36.1.453 15012509

[B66] Walters D. R. Newton A. C. Lyon G. D. (2005). Induced resistance: Helping plants to help themselves. *Biologist* 52 28–33. 10.7554/eLife.57389 33077025 PMC7679141

[B67] Williamson V. M. Roberts P. A. (2009). “Mechanisms and genetics of resistance,” in *Root-knot nematodes*, eds Perry R. N. Moens M. Starr J. (Wallingford: CABI Publishing), 301–325.

[B68] Zahradnikova H. Petrikova K. (2013). Nematocid effects of watercress (*Nasturtium officinaler*. Br.). *Acta Univ. Agric. Silvic Mendelianae Brun.* 61 233–236. 10.11118/actaun201361010233

